# Editorial: Exploring socio-cultural factors in human nutrition: the importance of multidimensional approaches

**DOI:** 10.3389/fpubh.2026.1977513

**Published:** 2026-10-09

**Authors:** Suman Chakrabarty, Heng Yaw Yong, Hanson Park

**Affiliations:** 1 Department of Anthropology, Mrinalini Datta Mahavidyapith, West Bengal State University, Kolkata, India; 2 Center for Transformative Nutrition & Health, Division of Nutrition and Dietetics, IMU University, Kuala Lumpur, Malaysia; 3 Department of Anthropology, Seoul National University, Gwanak-gu, Republic of Korea

**Keywords:** behavior, community type, culture, dietary knowledge, nutrition

## Introduction

Human nutrition is a multidimensional phenomenon shaped not only by biological requirements but also by social, cultural, economic, psychological, and environmental conditions. What people eat reflects the interaction of individual preferences and capabilities with income, food prices, social relationships, cultural norms, gender roles, migration, psychological wellbeing, physical activity, commercial influences, and food environments. Dietary behavior is therefore embedded within broader social and economic contexts that determine the opportunities, constraints, and meanings associated with food (1–3).

This perspective is increasingly important as populations experience interconnected challenges including undernutrition, micronutrient deficiencies, overweight and obesity, diet-related non-communicable diseases, food insecurity, and environmental pressures on food systems. These challenges frequently share common social and commercial determinants and may interact across the life course (4, 5). Globalization, urbanization, migration, changing employment patterns, and digitalization are also transforming food environments and dietary practices. Contemporary nutrition research must therefore examine not only what people eat, but why particular dietary behaviors emerge, under what circumstances they change, and how social and cultural environments influence their health consequences.

The contributions within this Research Topic, “*Exploring Socio-Cultural Factors in Human Nutrition: The Importance of Multidimensional Approaches*,” collectively demonstrate the limitations of approaches that interpret nutrition primarily through individual choice. Across diverse populations and settings, the studies illustrate how dietary practices are produced through interactions among social position, culture, gender, psychological experiences, economic resources, migration, lifestyle, and food environments.

## From individual choice to the social production of dietary behavior

Nutrition knowledge is important, but knowledge alone does not guarantee healthy dietary behavior. Food choices are also influenced by affordability, availability, convenience, social expectations, taste, identity, household responsibilities, and perceived behavioral control. Behavioral theories such as the Theory of Planned Behavior highlight the importance of attitudes, subjective norms, and perceived control in shaping dietary intentions (2).

This has important implications for nutrition education. Interventions based primarily on information provision may have limited effects when healthy foods are unaffordable, inaccessible, culturally unfamiliar, or incompatible with time and household constraints. Socioeconomic inequalities in diet are partly related to food prices and the resources available to households (6). Thus, nutrition education should be combined with approaches that improve the conditions in which dietary decisions are made.

## Culture, identity, and dietary change

Culture should not be treated merely as a demographic variable. Food is closely connected to identity, family, social belonging, tradition, religion, memory, and gender. Dietary change may therefore represent broader processes of social transformation.

Research on dietary acculturation demonstrates that migration and exposure to new food environments can produce complex combinations of retained, modified, and newly adopted dietary practices rather than a simple replacement of traditional diets (7). Similarly, culturally adapted interventions suggest that cultural context should be incorporated into intervention design rather than treated as a barrier to healthy eating (8). Traditional foods should consequently not be classified as inherently healthy or unhealthy without considering preparation methods, portion sizes, frequency, cultural meaning, and changing lifestyles.

## Socioeconomic position, food insecurity, and nutritional inequality

A multidimensional perspective also requires distinguishing dietary preference from dietary constraint. Individuals may value healthy eating but lack sufficient income, time, transportation, cooking facilities, or access to nutritious foods. Socioeconomic position therefore influences not only dietary behavior but also the range of feasible choices.

Food insecurity provides a particularly important example. It can contribute to dietary compromise, psychological stress, competing financial priorities, and difficulties in managing chronic diseases (9). Consequently, nutritional inequalities should not automatically be interpreted as failures of individual behavior. Programs such as medically tailored meals and other food-support interventions reflect an emerging recognition that improving nutrition may require addressing material and social conditions alongside individual behavior.

## Gender, psychological wellbeing, and food environments

Dietary practices are also embedded within gendered and psychological experiences. Household responsibilities, food preparation roles, workplace conditions, social support, stress, and perceived control can influence dietary and feeding practices. Psychological factors should therefore not be considered solely as individual characteristics; they may themselves be shaped by socioeconomic and social environments.

At the population level, food environments further influence what foods are accessible, affordable, promoted, and socially normalized (3). Digital technologies have expanded these environments through social media, targeted advertising, online food delivery, and digital marketing. Consequently, nutrition research should consider food environments as dynamic systems in which price, promotion, availability, working schedules, transport, digital exposure, and household resources interact.

## Toward an integrated biocultural and systems perspective

Taken together, the evidence supports an integrated model in which biological processes are embedded within behavioral, psychological, interpersonal, cultural, economic, institutional, and environmental systems. Such a perspective is consistent with ecological and systems approaches to nutrition and is particularly relevant to understanding the coexistence of undernutrition, micronutrient deficiencies, obesity, non-communicable diseases, and environmental pressures—the so-called double or triple burden of malnutrition (4, 5).

Environmental sustainability adds another dimension. Healthy dietary recommendations increasingly need to consider the environmental consequences of food production and consumption. The EAT–Lancet Commission emphasized the need for healthy diets within sustainable food systems (10). However, sustainability recommendations must remain sensitive to cultural, economic, and geographical contexts. A dietary pattern that is nutritionally desirable but inaccessible or culturally inappropriate is unlikely to generate equitable population-level improvements.

## Research, policy, and practice implications

Future research should move beyond predominantly cross-sectional associations toward longitudinal, life-course, and mixed-methods designs capable of examining how social and cultural conditions influence dietary trajectories. Explicit causal models linking structural determinants, food environments, psychosocial processes, dietary behavior, and biological outcomes are also needed. Greater attention should be given to inter-sectionality because socioeconomic position, gender, age, migration, ethnicity, and geography may interact to produce distinct nutritional vulnerabilities.

For policy, responsibility for healthy eating should not rest exclusively with individuals. Governments and institutions influence food prices, agricultural systems, marketing, urban planning, school and workplace food environments, social protection, and healthcare. Policies should therefore address structural determinants while improving physical and economic access to nutritious foods.

In clinical and public-health practice, nutritional assessment should incorporate relevant contextual factors such as food insecurity, social barriers, psychological stress, cultural preferences, and access to appropriate foods. Culturally responsive interventions should be developed collaboratively with communities and, where appropriate, linked with social services and other forms of support.

## Conclusion

The central contribution of a multidimensional approach to nutrition is the recognition that social and cultural factors are not merely associated with dietary behavior; they are part of the processes through which nutritional health is produced. Food practices emerge from interactions among biological needs, social relationships, cultural meanings, economic resources, psychological experiences, and changing food environments.

Improving nutritional health therefore requires more than providing individuals with information. It requires understanding why people eat what they eat, what makes particular choices possible or impossible, and how nutritional opportunities and risks are distributed across populations. Integrating biological, social, cultural, economic, and environmental perspectives can provide a stronger foundation for nutrition policies and interventions that are effective, equitable, culturally meaningful, and sustainable.
